# Environmentally Responsible and Cost-Effective Synthesis of the Antimalarial Drug Pyronaridine

**DOI:** 10.1021/acs.orglett.2c00944

**Published:** 2022-05-03

**Authors:** Joseph R. A. Kincaid, Rahul D. Kavthe, Juan C. Caravez, Balaram S. Takale, Ruchita R. Thakore, Bruce H. Lipshutz

**Affiliations:** Department of Chemistry & Biochemistry, University of California, Santa Barbara, California 93106 United States

## Abstract

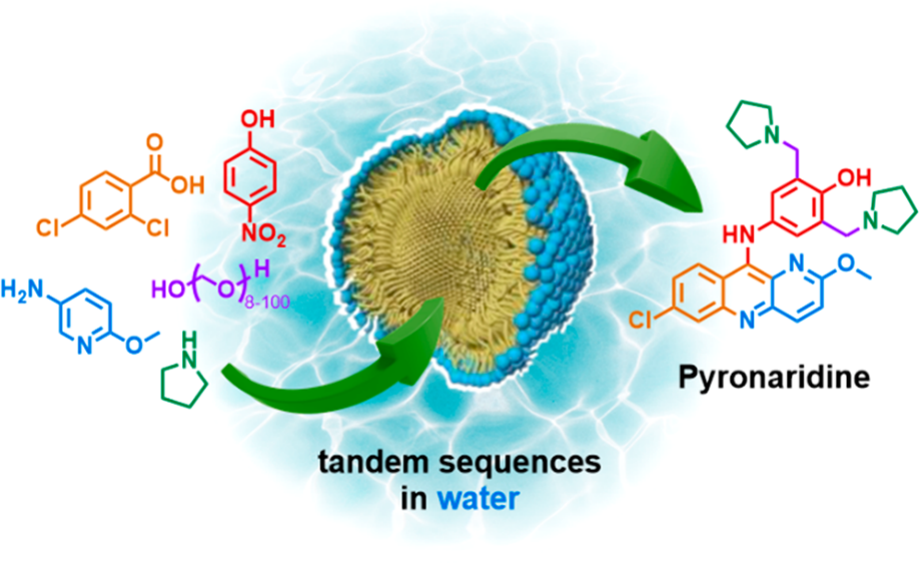

Two routes to the antimalarial drug Pyronaridine are described. The first is a linear sequence that includes a two-step, one-pot transformation in an aqueous surfactant medium, leading to an overall yield of 87%. Alternatively, a convergent route utilizes a telescoped three-step sequence involving an initial neat reaction, followed by two steps performed under aqueous micellar catalysis conditions affording Pyronaridine in 95% overall yield. Comparisons to existing literature performed exclusively in organic solvents reveal a 5-fold decrease in environmental impact as measured by E Factors.

The antimalarial drug Pyronaridine was developed in the 1970s and 1980s in response to the rise in chloroquine-resistant *Plasmodium*.^1^ In addition to effectively treating malaria, it showed significantly lower toxicity compared to chloroquine,^1b,2^ and when administered in combination with other antimalarials such as sulfadoxine and pyrimethamine little-to-no resistance to the drug was observed.^3^ Current research into repurposing pyronaridine for the treatment of nonmalarial parasitic diseases,^4^ cancer,^5^ and bacterial^6^ and viral^7^ infections has shown promise, including its potential in the ongoing fight against the SARS-CoV-2 virus responsible for the COVID-19 pandemic.^7b^ Nonetheless, its primary purpose remains as a treatment for malaria, especially in tropical regions of the world. However, from the perspective of pharmaceutical companies, justifying the sale of drugs to the developing world can be fiscally challenging;^8^ hence, the development of a synthetic route which minimizes cost is imperative to incentivize the production of Pyronaridine. Moreover, increasing pressure from governmental restrictions, such as the REACH regulation in the EU,^9^ requires chemical manufacturers to reduce their environmental footprint, providing additional incentive for the development of a greener route to Pyronaridine.
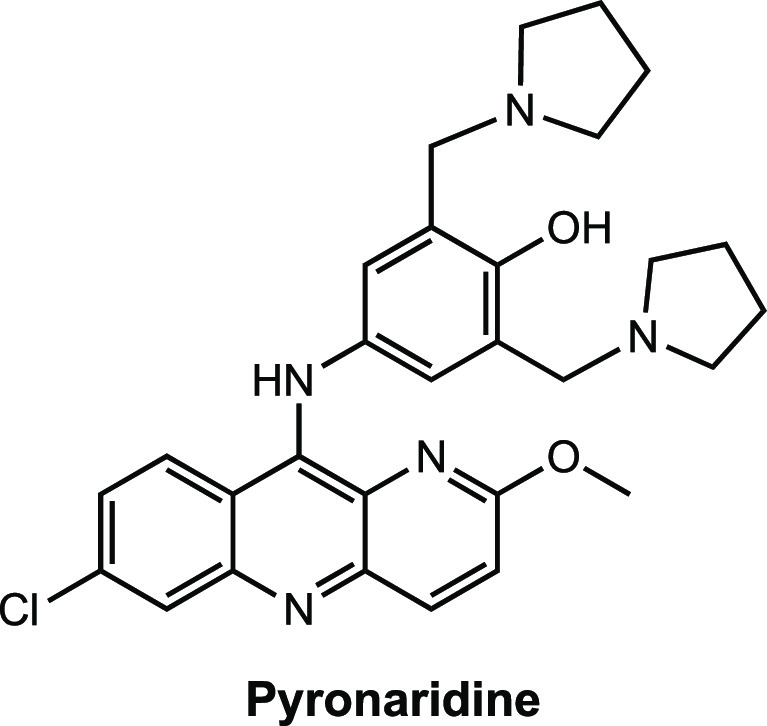


Fortunately, both aims are achievable by utilizing an environmentally responsible approach that relies on reactions run in Nature’s “solvent”: water.^10^ Such a “switch”^11^ is enabled using aqueous micellar catalysis, which simply involves the presence of an appropriately engineered and readily available micelle-forming nonionic surfactant that promotes solubilization of typically water-insoluble substrates.^12^ Furthermore, use of a common aqueous medium enables telescoping of reactions, thereby eliminating wasteful intermediate workups,^13^ while gaining in both time and pot economies.^14^ We now describe our recent efforts in collaboration with the Bill and Melinda Gates Foundation that have led to two inexpensive, scalable, and environmentally attractive routes to Pyronaridine.

## Overview

**Scheme 1:**
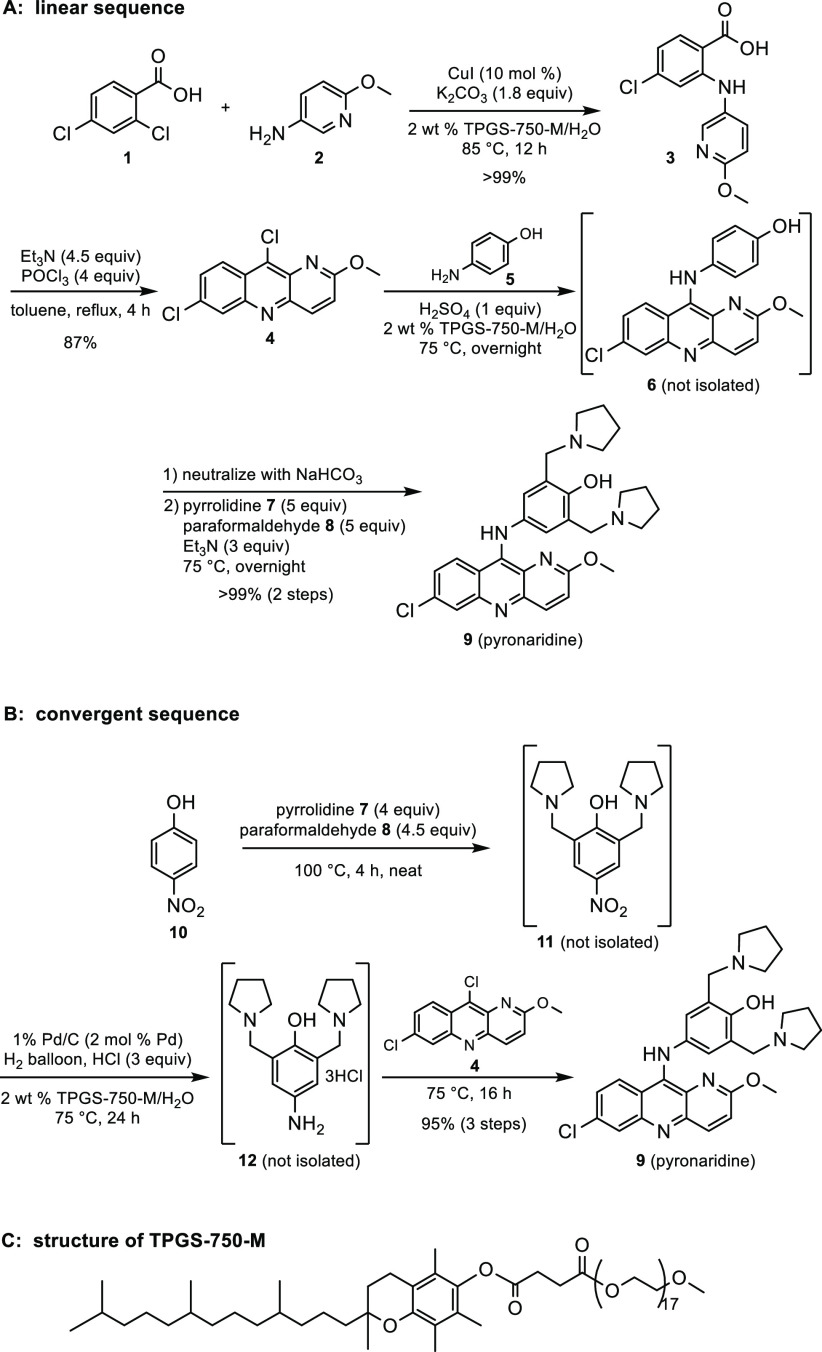
Overview of Linear and Convergent Routes to Pyronaridine

Both linear and convergent routes were developed for consideration toward scaling up a synthesis of Pyronaridine (Scheme 1A and B). Each shares the same initial Cu(I)-catalyzed Ullmann coupling between commercially available 2,4-dichlorobenzoic acid **1** and aminopyridine **2** to arrive at adduct **3**, run under aqueous micellar catalysis conditions derived from amphiphile TPGS-750-M^15^ (shown in Scheme 1C). Subsequent cyclization/deoxychlorination to afford the dichlorotricyclic heteroaromatic **4** required the use of especially water-sensitive POCl_3_ and was, therefore, carried out in recyclable toluene. The linear route continues directly from here via a two-step, one-pot acid-induced S_N_Ar involving **4** and *p*-aminophenol **5** leading to intermediate **6**. Without isolation, **6** undergoes a double Mannich-like reaction. Both reactions take place smoothly in aqueous solution to cleanly afford Pyronaridine (**9**).

Alternatively, the convergent route employs a three-step tandem sequence involving an initial double Mannich-like reaction using nitrophenol **10** as educt to install the two required methylpyrrolidine residues to arrive at **11**. In this case, the reaction could be performed in the complete absence of any reaction medium (i.e., under *neat* conditions). Nitro group reduction of **11** to **12** is then readily effected in aqueous surfactant solution. An acid-induced S_N_Ar reaction between **4** and **12** merges both components to afford Pyronaridine, **9**.

### Ullmann Coupling to Acid **3**

The Ullmann reaction between acid **1** and aminopyridine **2** to form **3** was performed in an aqueous solution of 2 wt % TPGS-750-M^15^ at a global concentration of 1 M. Purification involved acidification of the aqueous medium to pH 4.0, followed by collection of the precipitated product **3** via filtration. Copper salts were most efficiently removed by centrifugation rather than by suction filtration as the latter approach created a large amount of foam. Unreacted aryl acid **1** could be isolated by further acidification of the filtrate to pH 1, collection by filtration, and recrystallization from EtOH and water. Optimization studies involving the amount of catalytic copper and the role of the surfactant are shown in Table 1. Increasing the loading of CuI from 5 to 10 mol % led to a moderate increase in yield (entry 2). The importance of the surfactant was also apparent, as in its absence the yield was reduced accordingly (entries 3 and 4); thus, the conditions in entry 2 were selected. Some surfactant remained in the precipitated product, although the yields in entries 1 and 2 do not reflect this. Importantly, the product could be used without further purification and the residual surfactant did not impact the yield or purity of the subsequent cyclization/deoxychlorination step (87% overall yield for both steps, vide infra). It is noteworthy that product **3** was obtained with very high regioselectivity (i.e., no substitution at the *para*-position was observed by HPLC).

**Table 1:**
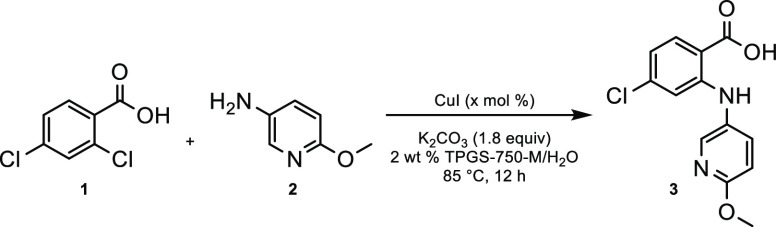
Optimization Studies of Ullmann Coupling

entry	CuI (mol %)	surfactant	isolated yield (%)
1	5	TPGS-750-M	91
2	10	TPGS-750-M	>99
3	5	none; on water	73
4	10	none; on water	82

### Cyclization/Deoxychlorination to Aryl Chloride **4**

**Figure 1:**
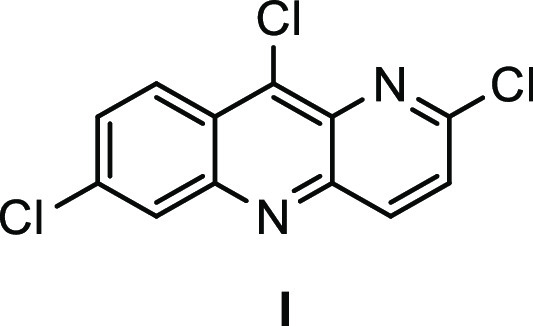
Potential impurity **I** resulting from demethylation.

Cyclization and concomitant deoxychlorination of compound **3** to form heteroaromatic **4** was achieved using POCl_3_. Et_3_N was included to prevent evolved HCl from demethylating the methoxy group and leading to impurity **I** (Figure 1) following subsequent deoxychlorination.^16^ Although water could not be used in this step due to the water-sensitive nature of POCl_3_, toluene served nicely and could be recovered following isolation of product **4**, thereby limiting organic waste generation. The product was obtained in 87% yield.

## Linear Sequence

### S_N_Ar Reaction to Phenol **6**

The S_N_Ar reaction between **4** and aminophenol **5** was performed under acidic conditions (pH = 1) and proceeded smoothly using the ideal equimolar quantities of each coupling partner. Product **6** precipitated from the reaction mixture and was obtained in quantitative yield. Following washing with water, the product was obtained in high purity.

### Mannich-like Reaction to Form Pyronaridine

Initial screening of the double Mannich-like reaction between **6** and excess (10 equiv) pyrrolidine **7** and paraformaldehyde **8** under micellar catalysis conditions provided Pyronaridine, **9**, in quantitative yield. Reducing the loading of pyrrolidine **7** and paraformaldehyde **8** from 10 to 4 equiv led to a precipitous drop in conversion, from 100% to 33%, due to poor solubility of educt **6**. That is, the reaction containing 10 equiv pyrrolidine begins as a homogeneous emulsion, and then soon thereafter, Pyronaridine slowly precipitated to form a free-flowing suspension over the course of the reaction. The reaction containing 4 equiv, however, did not afford an initial homogeneous mixture, and instead, amine **6** formed a clump, thereby limiting the extent of conversion. This suggested that the excess pyrrolidine was acting either as a cosolvent, a base, or a combination thereof. The addition of 10 v/v % 2-MeTHF as cosolvent containing 4 equiv of **7** and **8** did not improve the overall conversion. However, use of five equivalents each of **7** and **8** in combination with three equivalents of Et_3_N showed the same initial solubilization and eventual precipitation of **9**. In this manner, Pyronaridine could be isolated in quantitative yield.

### Two-Step, One-Pot Conversion of **4** to Pyronaridine

The S_N_Ar followed by the Mannich-like reactions could be performed in water in a one-pot fashion. An initial S_N_Ar reaction between **4** and **5** was performed to afford adduct **6**. The reaction mixture was then neutralized with NaHCO_3_, after which was added **7**, **8**, and Et_3_N. Pyronaridine **9** was thereby obtained in quantitative yield over both steps. The overall linear sequence, therefore, is performed in four individual steps in three pots. The overall efficiency has been increased from 43%^16^ to 87% using this process.

## Convergent Sequence

### Mannich-like Reaction to Form Nitroarene **11**

The Mannich-like reaction between *p*-nitrophenol **10**, pyrrolidine **7**, and paraformaldehyde **8** was performed under neat conditions. Following addition of **10** and **7** to the pot, the exotherm was effectively mitigated by slow portion-wise addition of **8** at 5 °C. Once all of the reagents had been added, the mixture was heated to 100 °C for 4 h. The remaining pyrrolidine was removed by codistillation with methanol, whereupon nitro compound **11** was obtained in quantitative yield. No over- or under-substitution was observed.

### Reduction of Nitroarene **11** to Aniline **12**

The nitro group reduction could be performed in aqueous surfactant solution using Pd/C and atmospheric hydrogen pressure.^17^ It was necessary to include HCl, as the free-base aniline is highly unstable. Because product **12** is water-soluble, isolation from the aqueous solution is not straightforward. Fortunately, it was found that the subsequent S_N_Ar reaction could be performed in a tandem fashion (see sequence below).

### S_N_Ar Reaction with **12** Leading to Pyronaridine

The S_N_Ar reaction was performed in 2 wt % aqueous TPGS-750-M under acidic conditions using a 1:1 ratio of coupling partners **4** and **12**. Pyronaridine **9** precipitated from the aqueous reaction mixture, and was isolated via centrifugation followed by washing the solid with water leading to the targeted drug in 78% isolated yield. Interestingly, this yield is lower than would be expected from the 3-step tandem sequence in which Pyronaridine is obtained in 95% yield (vide infra). This suggests that in situ formation of the aniline salt, as in the tandem sequence, is preferrable to direct use of the isolated trihydrochloride salt.

### Tandem Three-Step Sequence to Pyronaridine

Beginning with nitrophenol **10** and having heteroaromatic **4** in hand (vide supra), both pyrrolomethylene groups were installed to yield intermediate **11**. Stripping with methanol removed excess pyrrolidine, to which was then added an aqueous 2 wt % solution of TPGS-750-M. Introduction of HCl and Pd/C under an atmosphere of H_2_ led to nitro group reduction to give intermediate **12**. The Pd/C was then removed via filtration of the reaction mixture through a short plug of Celite. To this filtrate was then added heteroaryl chloride **4**, and the mixture was allowed to react at 75 °C for 16 h. Upon completion, the solution was basified to pH 8 with aqueous NH_4_OH and the precipitated Pyronaridine (**9**) was collected via filtration, washed with water, and isolated in an overall yield of 95% over three steps. Since Pyronaridine is offered as its tetraphosphate salt, opportunities for further purification exist, although they were not pursued in these studies.

The convergent process involves a three-step sequence from educt **10** that avoids isolation of intermediates and proceeds in an overall yield of 95%. Clearly, this approach has significant advantages that include: (1) avoidance of waste-generating organic solvents; (2) both time^14a^ and pot^14b^ economies, avoiding workup normally associated with each step; (3) the overall efficiency of the process has been increased from the literature value of 69%^18^ to 95%.

## E Factors

**Scheme 2:**
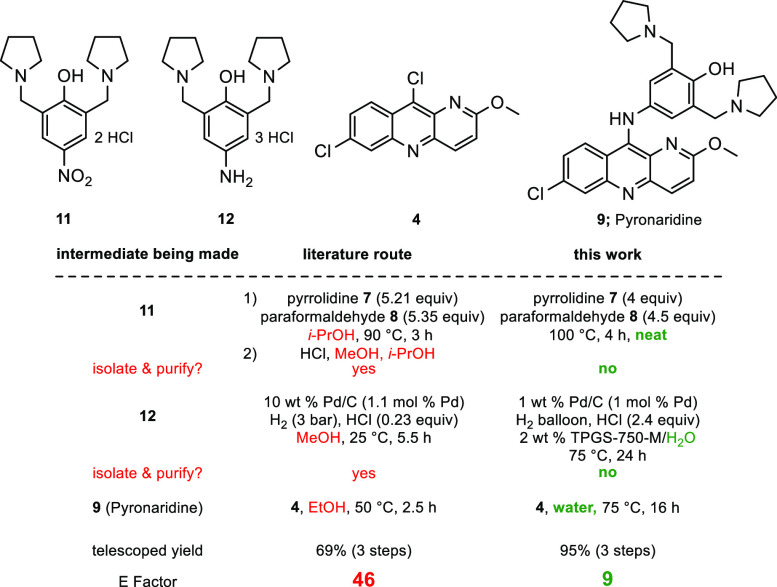
Comparisons with Existing Route^18^

To quantify the reduction in environmental impact relative to a literature protocol,^18^ an Environmental Factor (E Factor)^19^ was determined, calculated as the ratio of the mass of waste generated to the mass of product. This was evaluated for the three-step tandem sequence leading to Pyronaridine **9**, starting with *p*-nitrophenol **10** (Scheme 1B). This led to a 5-fold decrease in E Factor to a very low value of 9, exemplifying the environmental friendliness of the described sequence. Overall, some of the major comparisons between an existing literature route^18^ and the current, far greener synthesis are summarized in Scheme 2.

## Summary

Two economically attractive and environmentally responsible approaches to the synthesis of the antimalarial drug Pyronaridine have been disclosed which should reduce the cost barrier to its production for eventual distribution in various regions in the world. The convergent route appears to be the most effective in terms of both “greenness” and overall efficiency, but only by virtue of its scale up, currently underway, will its preferred status be confirmed.

## Supporting Information Available

Experimental procedures, optimization details, and analytical data (NMR, HPLC, and MS) (PDF)
