# Supplementary material for: Environmentally Responsible and Cost-Effective Synthesis of the Antimalarial Drug Pyronaridine
Source: Org Lett. 2022 May 3;24(18):3342–6. doi: 10.1021/acs.orglett.2c00944 (PMC9112334; doi:10.1021/acs.orglett.2c00944)
Supplement: Supplementary file 1 — ol2c00944_si_001.pdf [file ol2c00944_si_001.pdf]

Supporting Information

Environmentally Responsible and Cost-effective Synthesis of the Antimalarial Drug Pyronaridine

Joseph R. A. Kincaid, Rahul D. Kavthe, Juan C. Caravez, Balaram S. Takale,

Ruchita R. Thakore, and Bruce H. Lipshutz

Department of Chemistry and Biochemistry, University of California, Santa Barbara, CA 93106 USA

Phone : 805-893-2521
Fax : 805-893-8265
Email: lipshutz@chem.ucsb.edu
Website: https://lipshutz.chem.ucsb.edu/

Table of Contents

1. General Information 2
2. Synthetic Schemes 3
3. Procedures Common to Linear and Convergent Sequences 4
3.1 Ullmann Coupling 4
3.2 Deoxychlorination/Cyclization 4
4. Procedures for Linear Sequence 5
4.1 S_NAr Reaction 5
4.2 Mannich-Like Reaction 5
4.3 1-Pot S_NAr, then Mannich-Like Reaction 6
5. Procedures for Convergent Sequence 6
5.1 Mannich-Like Reaction 6
5.2 Nitro Group Reduction 7
5.3 S_NAr Reaction 7
5.4 Tandem Mannich, then Nitro Group Reduction, then S_NAr Reaction 8
6. E Factor Calculations 10
7. References 11
8. Experimental Data 12
9. ^1H, ^{13}C NMR Spectra of Synthesized Products 14

1. General Information

Surfactant Solution Preparation:

A 2 wt % TPGS-750-M/H_2O solution was prepared by dissolving TPGS-750-M in degassed HPLC grade water. TPGS-750-M[1] was made as described previously and is also commercially available. Reagents were purchased from Sigma-Aldrich, Combi-Blocks, Alfa Aesar, or Acros Organics.

Chromatography:

Silica gel TLC plates (UV 254 indicator, thickness 200 mm standard grade, glass backed and 230-400 mesh from Merck) were used. The developed TLC plate was analyzed by a UV lamp (254 nm). The plates were further analyzed with the use of an aqueous ceric ammonium molybdate stain or ethanolic vanillin and developed with a heat gun. All commercially available reagents were used without further purification. Flash chromatography was performed using Silicycle Silicaflash® P60 unbonded grade silica.

NMR:

^1H and ^{13}C NMR were recorded at 25 ºC on either an Agilent Technologies 400 MHz, a Bruker Avance III HD 400 MHz, or a Varian Unity Inova 600 MHz spectrometer in $CDCl_3$ or DMSO-d_6 with residual $CHCl_3$ (^1H = 7.26 ppm, ^{13}C = 77.16 ppm) or DMSO (^1H = 2.54 ppm, ^{13}C = 40.45 ppm) as the internal standard. Chemical shifts are reported in parts per million (ppm). The data presented will be reported as follows; chemical shift, multiplicity (s = singlet, bs = broad singlet, d = doublet, dd = doublet of doublet, t = triplet, q = quartet, quin = quintet, m = multiplet), coupling constant (if applicable), and integration.

HPLC:

HPLC analysis was performed on an Agilent 1220 series HPLC with an Agilent Poroshell HPH C18 column (4.6 x 50 mm, 2.7 µm). HPLC-grade solvents were obtained from Fischer Scientific.

2. Synthetic Schemes

Linear Sequence:

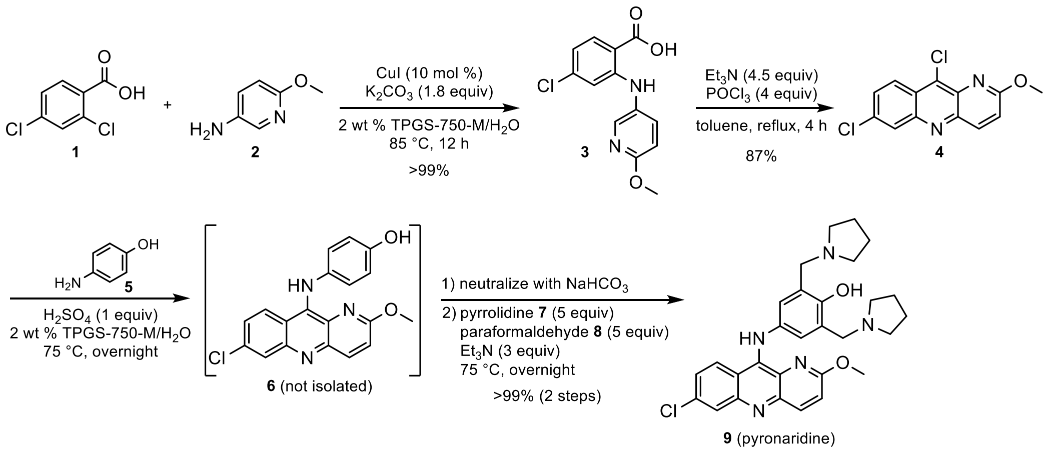

Convergent Sequence:

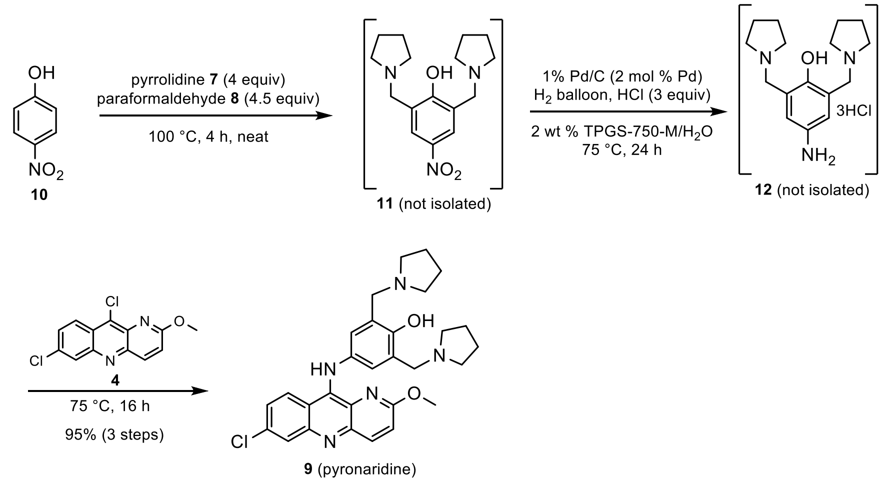

3. Procedures Common to Linear and Convergent Sequences

3.1 Ullmann Coupling

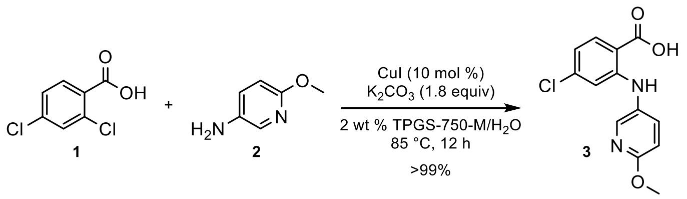

To a 50 mL cylindrical Schlenk flask equipped with a PTFE-coated magnetic stir bar was added 2,4-dichlorobenzoic acid **1** (1.2 equiv, 7.85 mmol, 1.50 g) and K_2CO_3 (1.8 equiv, 11.78 mmol, 1.627 g), then the flask was transferred to a glove box where CuI (10 mol %, 0.785 mmol, 150 mg) was added under argon. The side-arm of the tube was sealed with a rubber septum and the flask was removed from the glove box, then a solution of 2 wt % TPGS-750-M/H_2O (15.7 mL) was charged under a flow of argon and the mixture allowed to stir for 10 min at rt. The amine **2** (1 equiv, 6.54 mmol, 0.74 mL) was added slowly via syringe and the flask was sealed and allowed to stir at 85 °C for 12 h. Upon completion, the reaction mixture was transferred to a 15 mL centrifuge tube and centrifuged to remove the copper catalyst, after which the supernatant was transferred to a 250 mL round-bottom flask and acidified to pH 4.0 using a pH meter with 1 M aqueous HCl. Care was taken to not go below pH 4.0 as unreacted **1** begins to precipitate at pH 3.2. The product **3** precipitated at a grey-purple solid and was recovered by filtration, washed with water 3 times, and dried under vacuum at room temperature (1.8283 g, >99% yield).

CAUTION: The reaction evolves CO_2 and is heated in a closed vessel. Glassware must be rated to withstand high-pressure reactions and should be inspected for cracks or fractures prior to use.

3.2 Deoxychlorination/Cyclization

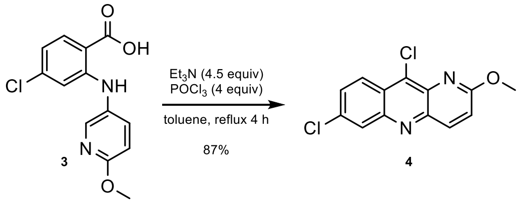

To a flame-dried 2 dr vial equipped with a PTFE-coated magnetic stir bar was added aminobenzoic acid derivative **3** (1 equiv, 1 mmol, 278.7 mg) and the vial was flushed with argon, then charged with anhydrous toluene (2 mL) under a flow of argon, followed by Et_3N (4.5 equiv, 4.5 mmol, 627 μL) and $POCl_3$ (4 equiv, 4 mmol, 374 μL) and the flask was sealed and allowed to stir at 110 °C

for 4 h. Upon completion, the reaction mixture was transferred dropwise to a beaker containing 30 mL of ice water and 5 mL of 30% aqueous NH_4OH with strong stirring. The product **4** precipitated as a grey solid which was recovered via filtration, washed with water three times, and dried under vacuum at room temperature (242.8 mg, 87% yield).

4. Procedures for Linear Sequence

4.1 SNAr Reaction

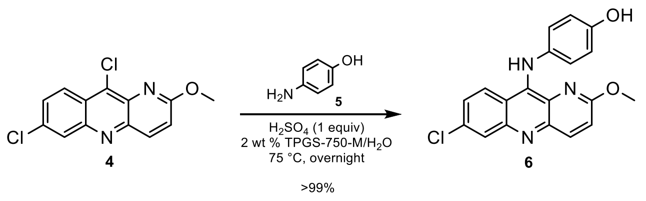

To a 1 dr vial equipped with a PTFE-coated magnetic stir bar was added aryl chloride **4** (1 equiv, 1.25 mmol, 349 mg), *p*-nitrophenol **5** (1 equiv, 1.25 mmol, 136 mg), conc. H_2SO_4 (1 equiv, 1.25 mmol, 67 µL), and a solution of 2 wt % TPGS-750-M/H_2O (2.5 mL), then the vial was sealed and allowed to stir at 75 °C overnight. Upon completion, the orange precipitate of product **6** was collected via centrifugation and washed with water three times by repeated resuspension in DI water and centrifugation, then dried under vacuum at room temperature (436.8 mg, >99% yield).

4.2 Mannich-like Reaction

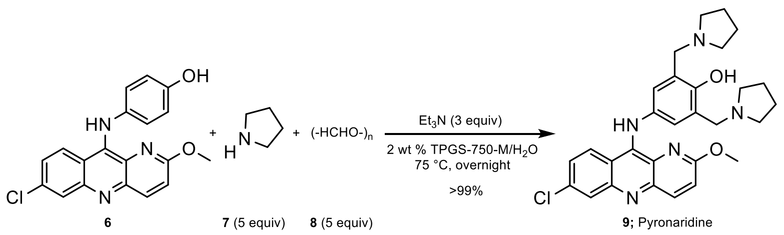

To a 1 dr vial equipped with a PTFE-coated magnetic stir bar was added phenol derivative **6** (1 equiv, 0.25 mmol, 88 mg), paraformaldehyde **8** (5 equiv, 1.25 mmol, 37.5 mg), pyrrolidine **7** (5 equiv, 1.25 mmol, 103 µL), a solution of 2 wt % TPGS-750-M/H_2O (0.4 mL), and Et_3N (3 equiv, 0.75 mmol, 105 µL), then the vial was sealed and allowed to stir at 75 °C overnight. Upon completion, the orange precipitate of pyronaridine **9** was collected via centrifugation and washed with water three times by repeated resuspension in DI water and centrifugation, then dried under vacuum at room temperature (87.7 mg, >99% yield).

CAUTION: The addition of pyrrolidine is exothermic. On larger scales it may be necessary to add 2 wt % TPGS-750-M/H_2O prior to the addition of pyrrolidine to control the exotherm.

4.3 1-Pot S_NAr, then Mannich-like Reaction

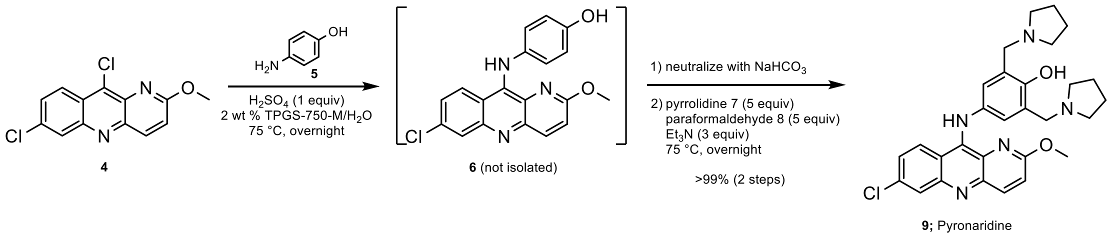

To a 1 dr vial equipped with a PTFE-coated magnetic stir bar was added aryl chloride **4** (1 equiv, 0.5 mmol, 140 mg), *p*-nitrophenol **5** (1 equiv, 0.5 mmol, 55 mg), conc. H_2SO_4 (1 equiv, 0.5 mmol, 27 µL), and a solution of 2 wt % TPGS-750-M/H_2O (1.0 mL), then the vial was sealed and allowed to stir at 75 °C overnight. Upon completion of the S_NAr reaction, the mixture was cooled to rt and neutralized to pH 7 with $NaHCO_3$, then to the vial was added paraformaldehyde **8** (5 equiv, 2.5 mmol, 75 mg), pyrrolidine **7** (5 equiv, 2.5 mmol, 205 µL), and Et_3N (3 equiv, 1.5 mmol, 210 µL), then the vial was sealed and allowed to stir at 75 °C overnight. Upon completion of the Mannich-like reaction, the orange precipitate of pyronaridine **9** was collected via centrifugation and washed with water five times by repeated resuspension in DI water and centrifugation, then dried under vacuum at room temperature (129.1 mg, >99% yield).

5. Procedures for Convergent Sequence

5.1 Mannich-like Reaction

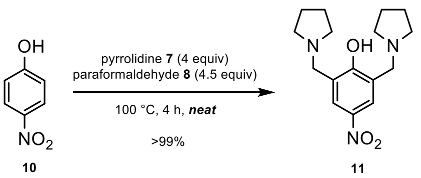

Neat Conditions: To a 2-dram vial containing a PTFE-coated magnetic stir bar was added paraformaldehyde **7** (4.5 equiv, 4.5 mmol, 135 mg), pyrrolidine **8** (4 equiv, 4 mmol, 328 µL), and *p*-nitrophenol **10** (1 equiv, 1 mmol, 139.1 mg). The vial was capped and stirred in an aluminum heating block on a heating stir plate at 100 °C for 4 h. Upon completion, methanol was added and dissolved by stirring, then removed *in vacuo* to remove both methanol and excess pyrrolidine and

yield the product as a yellow oil that was observed to crystallize on standing for several days. To obtain pure product for characterization purposes (i.e., free of residual methanol and trace pyrrolidine), the product was purified via flash chromatography on neutral alumina using a gradient of 1-5% MeOH/CH_2Cl_2 (303.8 mg, >99% yield). Column purification was not necessary for the 3-step, 1-pot sequence.

5.2 Nitro Group Reduction

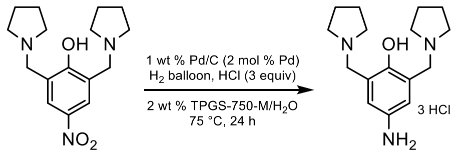

11 **12**

See tandem sequence below for aqueous protocol. The water-soluble trihydrochloride salt is not isolable from water. Attempts to isolate the free-base aniline were unsuccessful as it is highly unstable and degrades within minutes. The isolated trihydrochloride salt was prepared in methanol according to a literature protocol.[2]

5.3 S_NAr Reaction

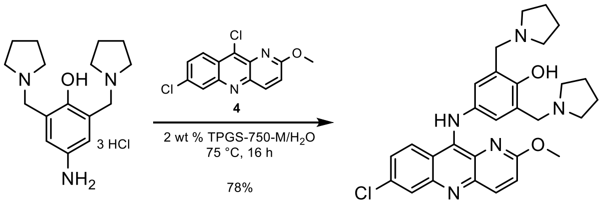

12 **9**; Pyronaridine

To a 1 dr vial equipped with a PTFE-coated magnetic stir bar was added aryl chloride **4** (1 equiv, 0.25 mmol, 70 mg), aminophenol trihydrochloride derivative **12** (1 equiv, 0.25 mmol, 96 mg), and 2 wt % TPGS-750-M/H_2O (0.5 mL). The vial was capped and stirred at 75 °C for 12 h. Upon completion, the reaction mixture was neutralized with aqueous 30% NH_4OH (ca. 1 mL) and the resulting precipitate was filtered to afford pyronaridine **9** as an orange solid (101.0 mg, 78% yield).

5.4 Tandem Mannich, then Nitro Group Reduction, then S_NAr Reaction

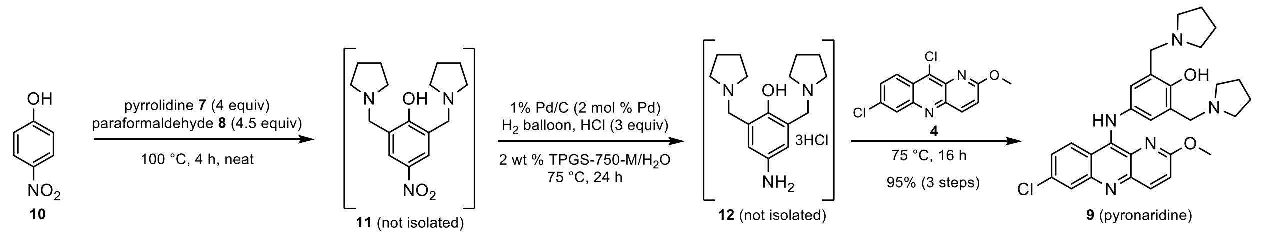

To a 1 dr vial equipped with a PTFE-coated magnetic stir bar was added nitrophenol **10** (1 equiv, 0.25 mmol, 35 mg) and pyrrolidine **7** (4 equiv, 1 mmol, 82 µL), then paraformaldehyde **8** was added portion-wise to limit the exotherm (4.5 equiv, 1.125 mmol, 35 mg), then the vial was capped and the mixture was stirred at 100 °C in an aluminum heating block on a heating stir plate for 4 h. Upon completion, methanol was added and dissolved by stirring, then removed *in vacuo* to remove both methanol and excess pyrrolidine. To the residue was added 2 wt % TPGS-750-M/H_2O (0.5 mL), conc. HCl (3 equiv, 0.75 mmol, 63 µL), and 1 wt % Pd/C (2 mol %, 53.2 mg). The vial was capped with a rubber septum and fitted with a balloon full of H_2 gas, then the reaction mixture was stirred at 75 °C for 16 h. Upon completion, the reaction mixture was filtered through a short plug of diatomaceous earth to remove Pd/C and the filtrate was collected in a separate 1 dr vial with a PTFE-coated magnetic stir bar. To this vial was added aryl chloride **4** (1 equiv, 0.25 mmol, 70 mg), then the vial was capped and allowed to stir at 75 °C for 16 h. Upon completion, the reaction mixture was cooled to room temperature and neutralized with aqueous 30% NH_4OH (ca. 1 mL) and the resulting precipitate was collected via filtration and washed with water to afford pyronaridine **9** as an orange solid (122.8 mg, 95% yield), 95.4% purity by HPLC.

HPLC analysis of **9** from 3-step tandem sequence:

A = 0.1 % formic acid in 5 mM aqueous ammonium acetate

B = 0.1 % formic acid in acetonitrile

HPLC Method

time (min)	flow rate (mL/min)	%A	%B
0	0.5	95	5
2	1	95	5
4.5	1	60	40
7	1	30	70
12	1	10	90
15	1	10	90
15.1	1	95	5
20	1	95	5

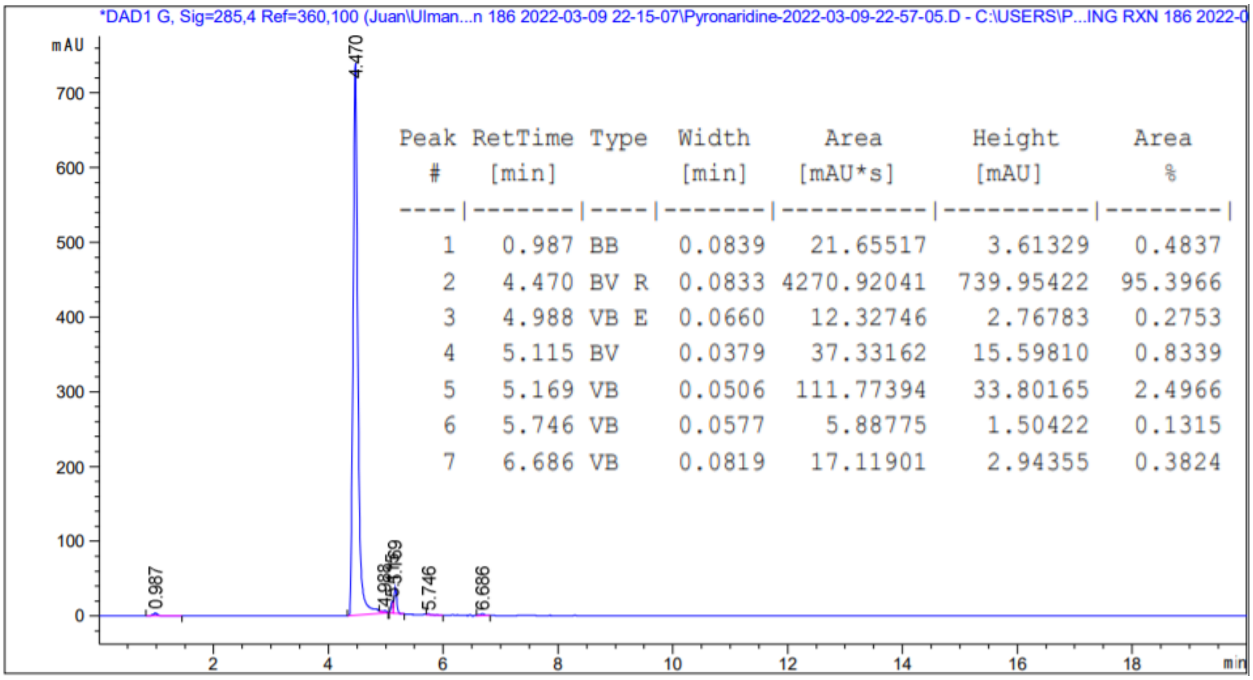

Peak #	RetTime [min]	Type	Width [min]	Area [mAU*s]	Height [mAU]	Area %
1	0.987	BB	0.0839	21.65517	3.61329	0.4837
2	4.470	BV R	0.0833	4270.92041	739.95422	95.3966
3	4.988	VB E	0.0660	12.32746	2.76783	0.2753
4	5.115	BV	0.0379	37.33162	15.59810	0.8339
5	5.169	VB	0.0506	111.77394	33.80165	2.4966
6	5.746	VB	0.0577	5.88775	1.50422	0.1315
7	6.686	VB	0.0819	17.11901	2.94355	0.3824

In cases where nitro compound **11** is not fully reduced, aryl chloride **4** cannot be completely consumed, and unreacted material will be partially demethylated to form impurity **II** under the acidic S_NAr conditions. These impurities can be removed by conversion of the crude material to pyronaridine tetraphosphate according to the protocol outlined by Liu, et al.[2] Quantification of impurity **II** by HPLC and NMR analyses was impracticable due to the compound's insolubility in most solvents. In leiu of these, TLC proved to be a sufficient qualitative method for determining its absence in the final product.

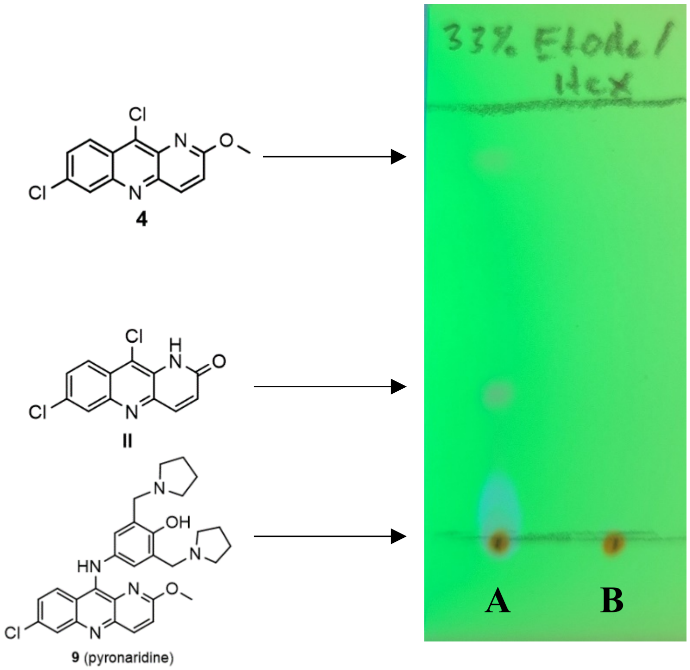

Lane A: Crude product from 3-step tandem sequence involving an incomplete nitro reduction

Lane B: Pyronaridine freebased from the tetraphosphate salt synthesized from material shown in lane A

6. E Factor Calculations

Masses of waste and product are normalized to 1 mmol of starting material for each step

This work:

Mannich-like reaction:

Excess pyrrolidine = 0.0440 g

Excess paraformaldehyde = 0.0232 g

Methanol = 0.4898 g

Nitro reduction:

2 wt % TPGS-750-M = 2.0 g

1 wt % Pd/C = 0.1064 g

S_NAr reaction:

NH_4OH = 1.76 g

Total waste = 4.5298 g

Total product = 0.4931 g

E Factor = 4.5298 / 0.4931 = 9

Literature method:[2]

Mannich-like reaction:

Excess pyrrolidine = 0.2284 g

Excess paraformaldehyde = 0.1006 g

Isopropanol = 0.8850 g

Dichloromethane = 0.6998 g

Water = 0.3521 g

Methanol = 0.8785 g

Nitro reduction:

Wet Pd/C = 0.0381 g

Water = 0.0063 g

Methanol = 1.5212 g

Ethanol = 1.1046 g

Ethyl acetate = 4.2116 g

S_NAr reaction:

Ethanol = 2.5270 g

Water = 3.4965 g

$NaOH/H_2O$ = 0.4272 g

Total waste = 16.4769 g

Total product = 0.3575 g

E Factor = 16.4769 / 0.3575 = 46

7. References

1. Lipshutz, B. H.; Ghorai, S.; Abela, A. R.; Moser, R.; Nishikata, T.; Duplais, C.; Krasovskiy, A.; Gaston, R. D.; Gadwood, R. C. *J. Org. Chem.* **2011**, *76*, 4379–4391.
2. Liu, Y.; Zhang, Z.; Wu, A.; Yang, X.; Zhu, Y.; Zhao, N. *Org. Process Res. Dev.* **2014**, *18*, 349-353.
3. Lee, D. W.; Lee, S. K.; Cho, J. H.; Yoon, S. S. *Bull. Korean Chem. Soc.* **2014**, *35*, 521-524.

8. Experimental Data

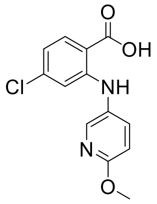

4-Chloro-2-((6-methoxypyridin-3-yl)amino)benzoic acid (3): ^{1}H NMR (600 MHz, $CDCl_3$) δ 9.17 (s, 1H), 8.12 (d, J = 2.7 Hz, 1H), 7.95 (d, *J* = 8.6 Hz, 1H), 7.52 (dd, *J* = 8.7, 2.8 Hz, 1H), 6.84 (d, *J* = 8.7 Hz, 1H), 6.77 (d, *J* = 2.0 Hz, 1H), 6.70 (dd, *J* = 8.6, 2.0 Hz, 1H), 3.99 (s, 3H). **R_f:** 0.47 (10% MeOH/CH_2Cl_2). Spectral data matched those previously reported.[2]

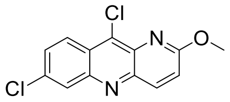

7,10-Dichloro-2-methoxybenzo[b][1,5]naphthyridine (4): ^{1}H NMR (400 MHz, $CDCl_3$) δ 8.37 (d, *J* = 9.2 Hz, 1H), 8.25 (d, *J* = 9.2 Hz, 1H), 8.17 (d, *J* = 2.1 Hz, 1H), 7.58 (dd, *J* = 9.2, 2.0 Hz, 1H), 7.25 (d, *J* = 9.1 Hz, 1H), 4.20 (s, 3H). **^{13}C NMR (100 MHz, $CDCl_3$) δ** 185.8, 162.7, 147.1, 144.6, 140.4, 138.6, 135.9, 135.6, 128.8, 128.4, 126.2, 125.2, 120.5, 54.5. **R_f:** 0.31 (5% EtOAc/hexanes). Spectral data matched those previously reported.[2]

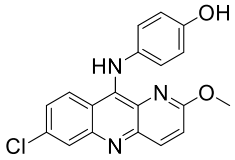

4-((7-Chloro-2-methoxybenzo[b][1,5]naphthyridin-10-yl)amino)phenol (6): ^{1}H NMR (400 MHz, DMSO-*d*$_6$) δ 9.40 (s, 1H), 9.02 (s, 1H), 8.21 (d, *J* = 9.2 Hz, 1H), 7.93 (d, *J* = 2.2 Hz, 1H), 7.75 (d, *J* = 9.4 Hz, 1H), 7.31 (d, *J* = 9.2 Hz, 1H), 7.20 (dd, *J* = 9.4, 2.2 Hz, 1H), 7.09 – 7.00 (m, 2H), 6.81 – 6.73 (m, 2H), 3.95 (s, 3H). **^{13}C NMR (126 MHz, DMSO-*d*$_6$) δ** 159.2, 154.6, 148.2, 144.3, 142.5, 140.3, 134.6, 133.5, 127.6, 126.7, 125.2, 122.9, 118.9, 115.6, 114.9, 53.7. **R_f:** 0.46 (0.5% Et_3N/9.5% MeOH/90% CH_2Cl_2). Spectral data matched those previously reported.[3]

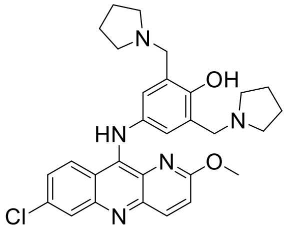

4-((7-Chloro-2-methoxybenzo[b][1,5]naphthyridin-10-yl)amino)-2,6-bis(pyrrolidin-1-ylmethyl)phenol (9): ^{1}H NMR (600 MHz, $CDCl_3$) δ 8.40 (s, 1H), 8.20 (d, J = 9.1 Hz, 1H), 7.99 (d, J = 2.1 Hz, 1H), 7.57 (d, J = 9.4 Hz, 1H), 7.20 (d, J = 9.1 Hz, 1H), 6.97 (s, 2H), 6.94 (dd, J = 9.4, 2.2 Hz, 1H), 4.09 (s, 3H), 3.73 (s, 4H), 2.68-2.56 (m, 8H), 1.89-1.78 (m, 8H). **^{13}C NMR (126 MHz, $CDCl_3$) δ** 160.4, 154.4, 149.3, 144.8, 142.6, 140.6, 135.0, 133.6, 128.4, 128.2, 127.1, 124.8, 124.2, 123.4, 119.3, 114.7, 56.5, 54.1, 54.0, 23.8. **R_f:** 0.20 (0.5% Et_3N/9.5% MeOH/90% CH_2Cl_2). Spectral data matched those previously reported.[3]

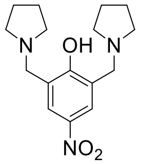

4-Nitro-2,6-bis(pyrrolidin-1-ylmethyl)phenol (11): ^{1}H NMR (400 MHz, $CDCl_3$) δ 8.0 (s, 2H), 3.79 (s, 4H), 2.67 - 2.60 (m, 8H), 1.89 - 1.80 (m, 4H), 3.45 (m, 8H). **^{13}C NMR (100 MHz, $CDCl_3$) δ** 163.6, 139.1, 124.2, 124.0, 56.0, 53.8, 23.6. **R_f:** 0.45 (5% MeOH/CH_2Cl_2). Spectral data matched those previously reported.[2]

9. ^{1}H, ^{13}C NMR Spectra of Synthesized Products

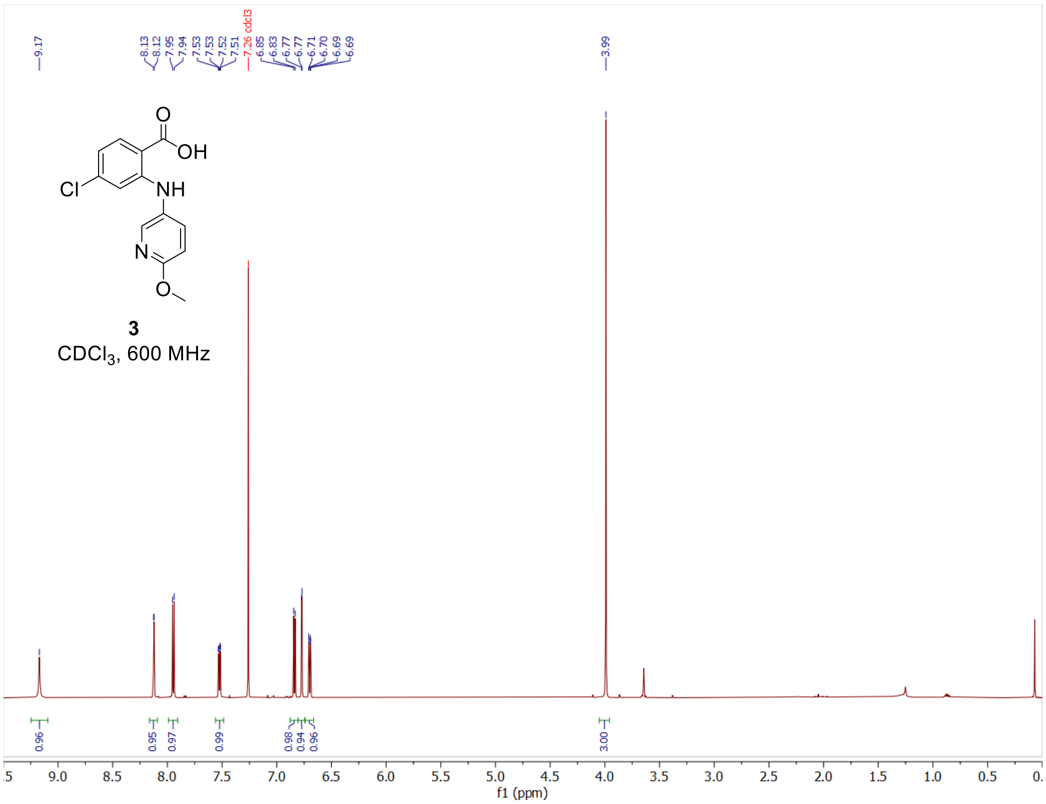

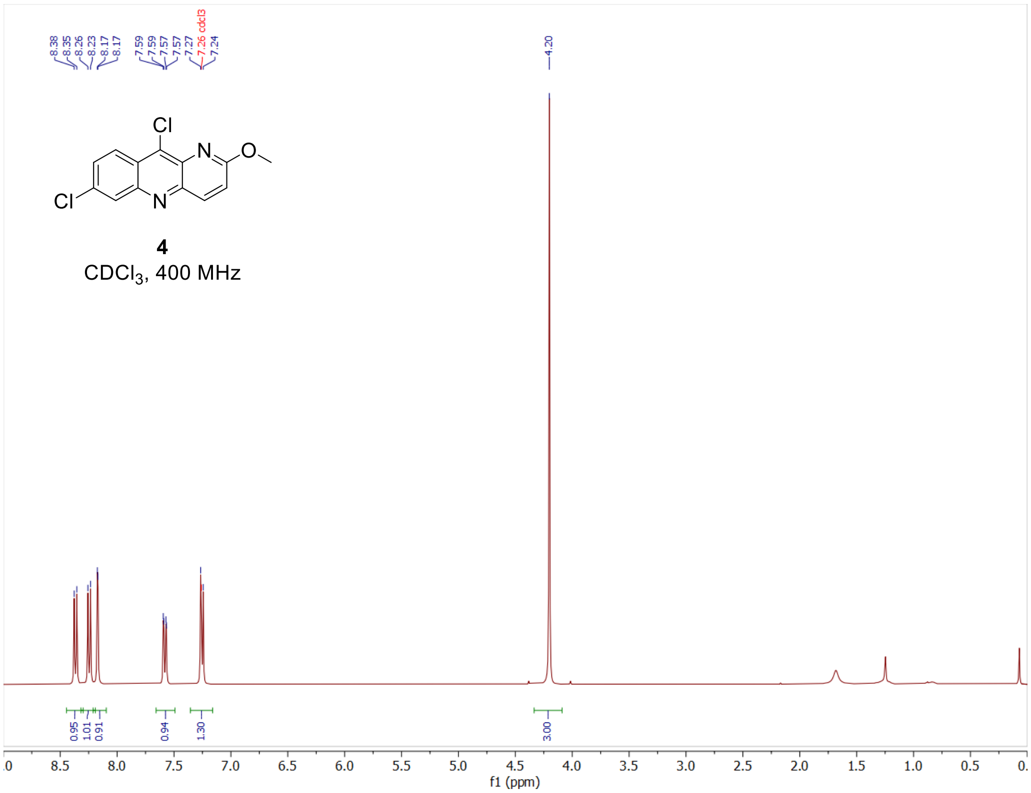

Cl
N
O
Cl
N
4
CDCl3, 400 MHz
f1 (ppm)

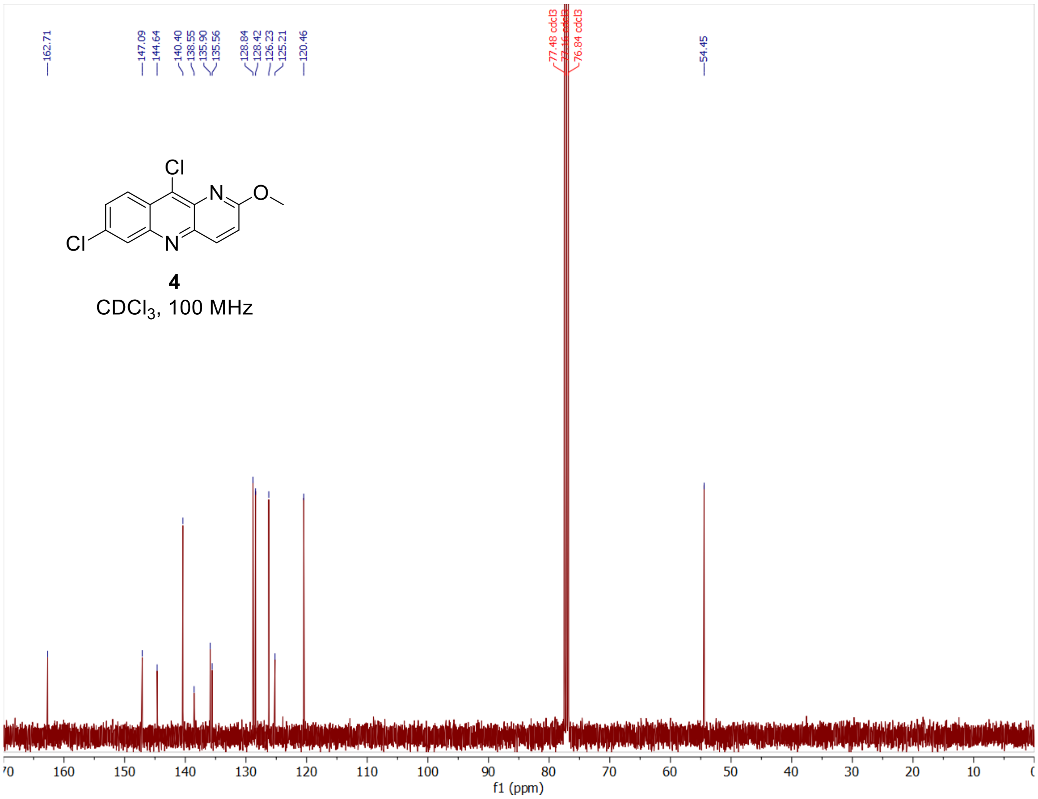

Cl
N
O
Cl
N
4
CDCl3, 100 MHz
f1 (ppm)

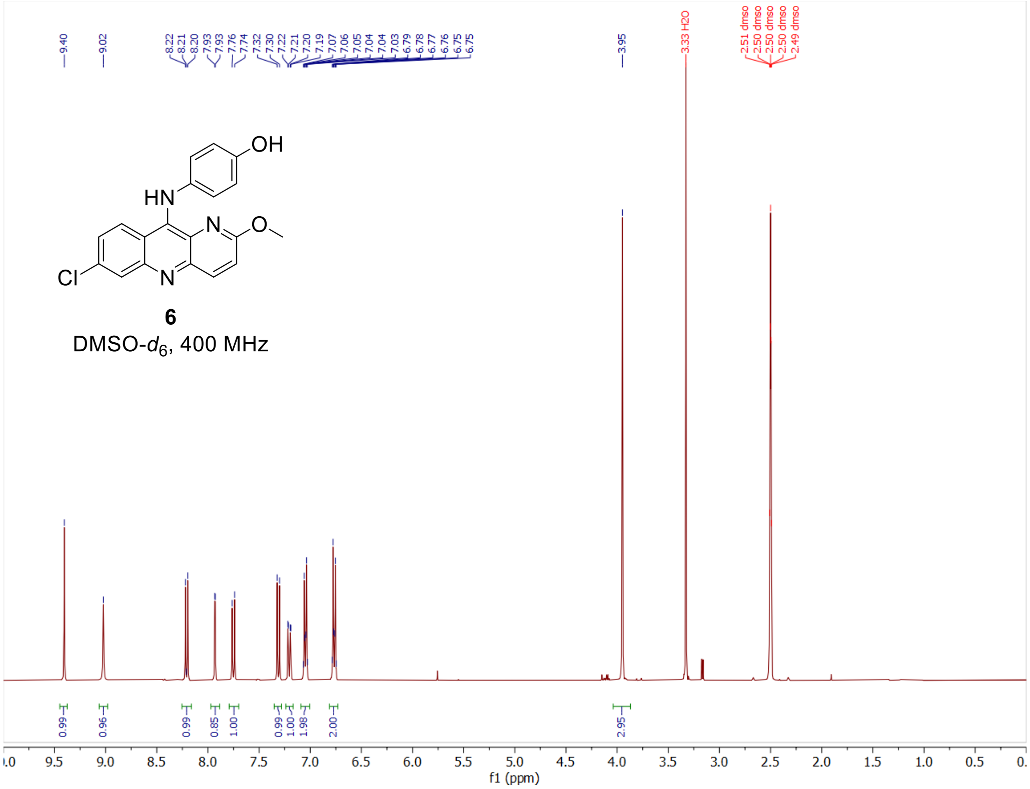
6
DMSO-d6, 400 MHz
f1 (ppm)

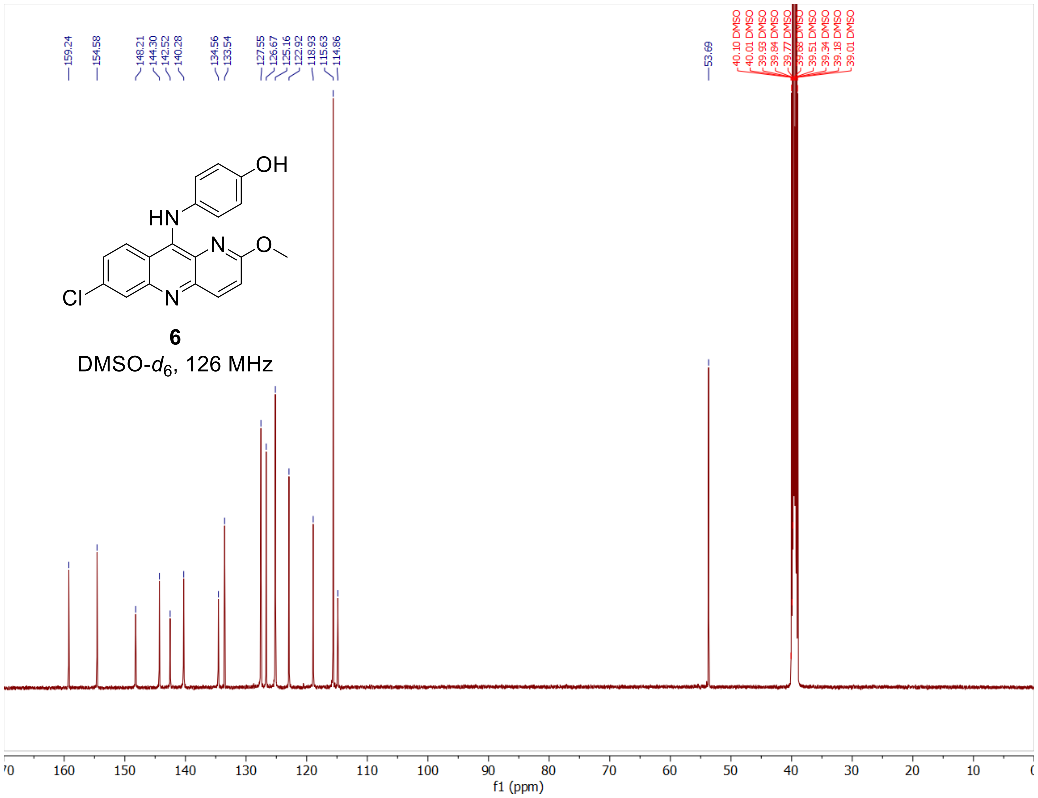
6
DMSO-d6, 126 MHz
f1 (ppm)

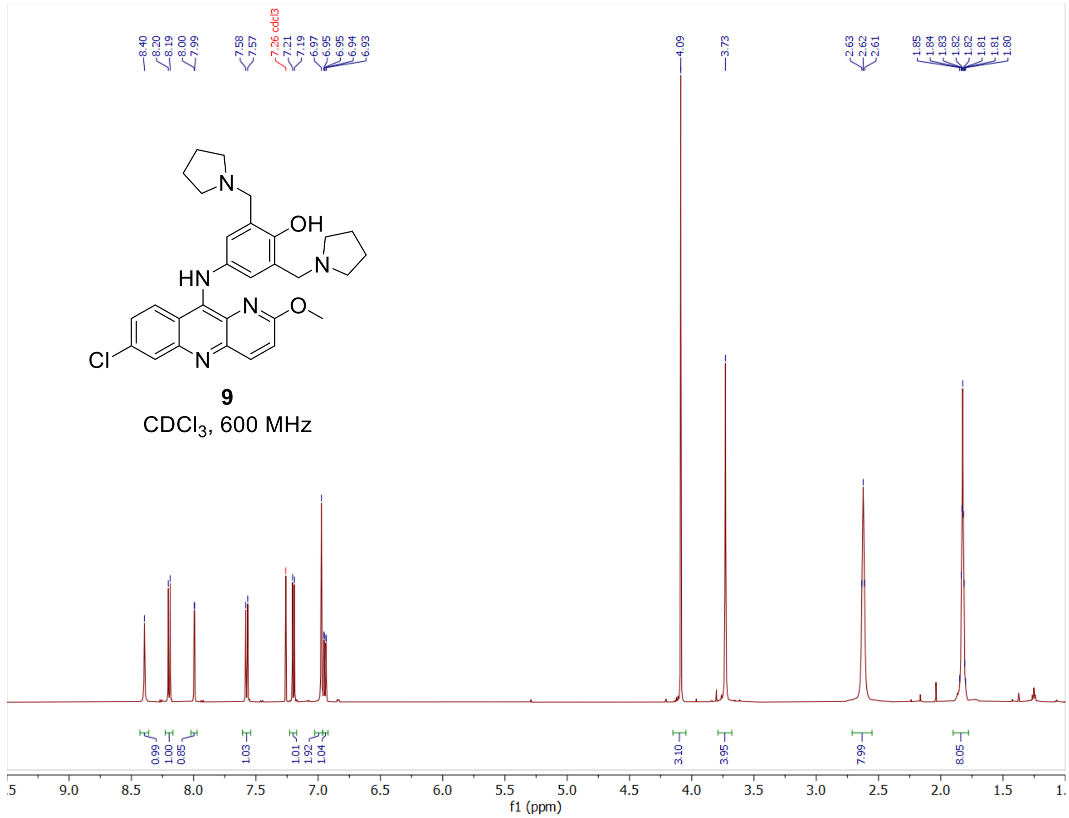

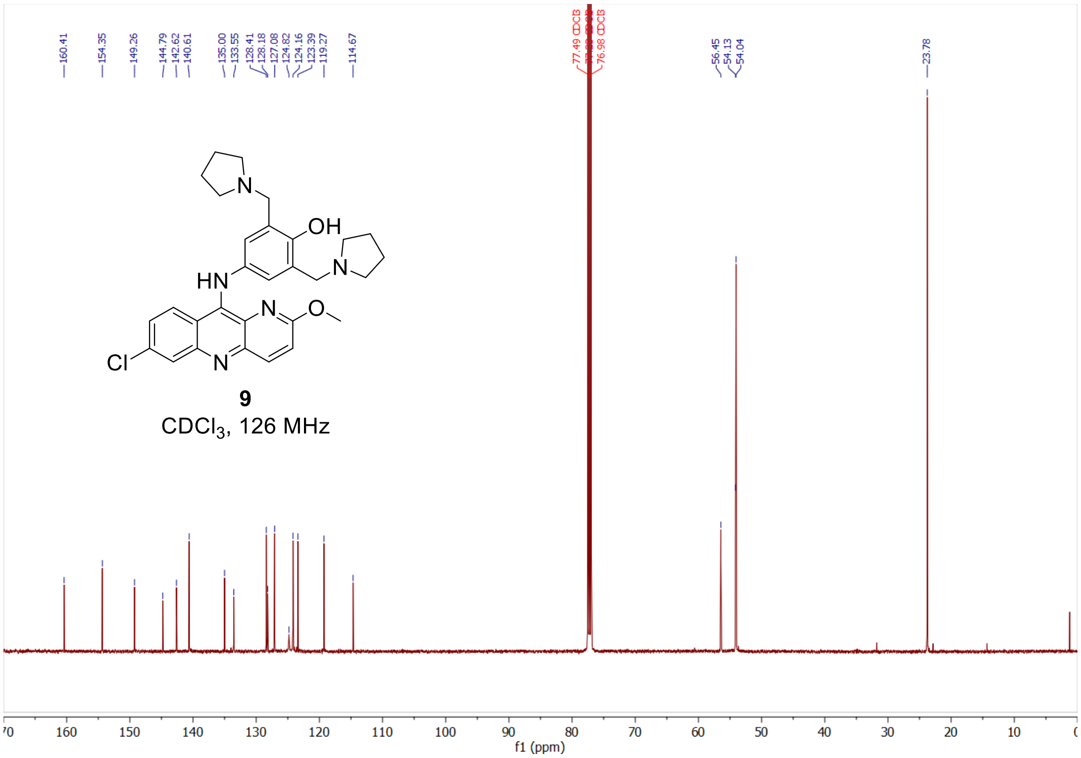

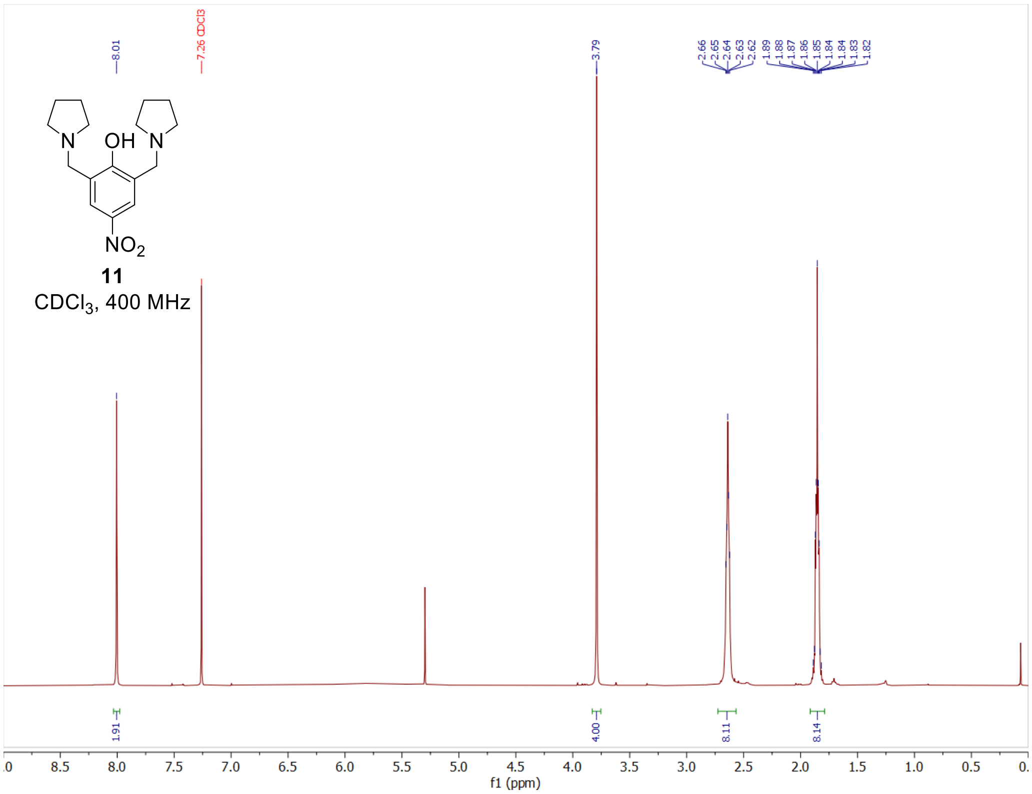
8.01
7.26 CDCl3
3.79
2.66
2.65
2.64
2.63
2.62
1.89
1.88
1.87
1.86
1.85
1.84
1.84
1.83
1.82
OH
N
N
NO2
11
CDCl3, 400 MHz
1.91
4.00
8.11
8.14
f1 (ppm)

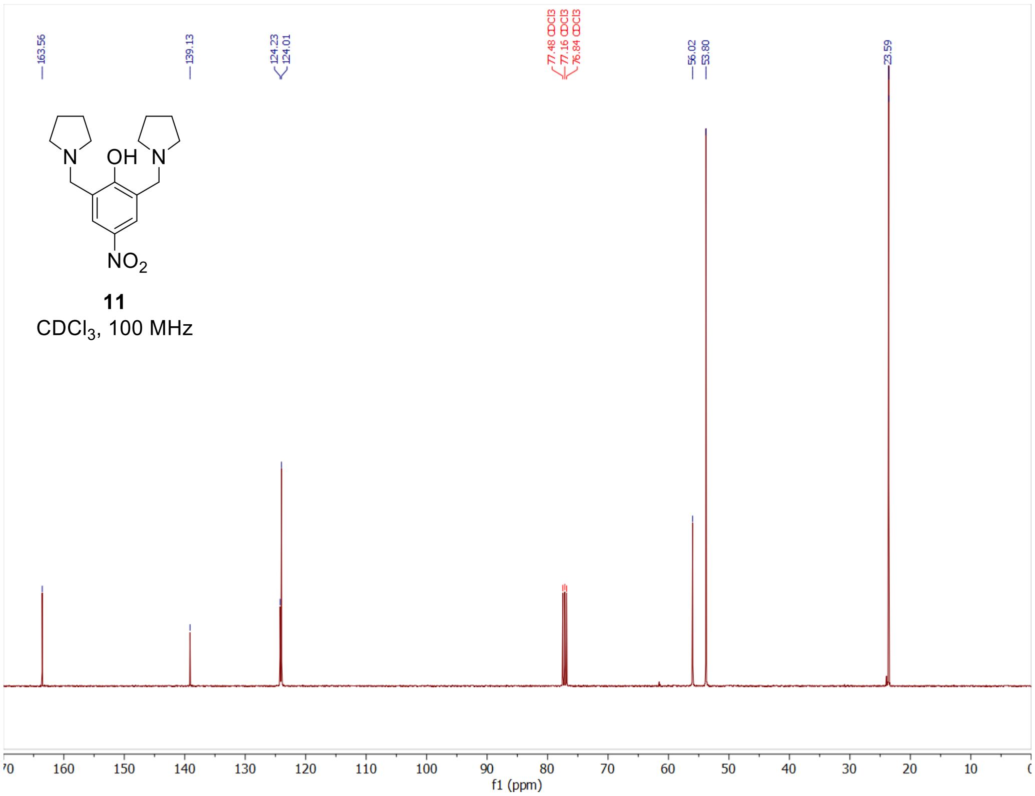
163.56
139.13
124.23
124.01
77.48 CDCl3
77.16 CDCl3
76.84 CDCl3
56.02
53.80
23.59
OH
N
N
NO2
11
CDCl3, 100 MHz
f1 (ppm)
